# Implementation Outcomes of Low Threshold Care for Persons with Opioid Use Disorders

**DOI:** 10.1007/s11606-025-10112-9

**Published:** 2026-01-14

**Authors:** Wayne Kepner, Hannah Cheng, Berkeley Franz, Andrea Jakubowski, Margaret Lowenstein, Elena Rosenberg-Carlson, Mark McGovern

**Affiliations:** 1 Division of Primary Care and Population Health, Department of Medicine, Stanford University School of Medicine, Palo Alto, CA USA; 2 Stanford Center for Dissemination and Implementation, Stanford University School of Medicine, Palo Alto, CA USA; 3 Department of Medical Social Sciences, Feinberg School of Medicine, Northwestern University, Chicago, IL USA; 4 Ohio University Heritage College of Medicine, Institute to ADVANCE Health Equity, Athens, OH USA; 5 Division of General Internal Medicine, Montefiore Medical Center/Albert Einstein College of Medicine, Bronx, NY USA; 6 Division of General Internal Medicine, University of Pennsylvania Perelman School of Medicine, Philadelphia, PA USA; 7 Department of Psychiatry and Behavioral Sciences, Stanford University School of Medicine, 1070 Arastradero Rd, Stanford, CA 94305 USA

**Keywords:** buprenorphine, implementation science, low threshold care, medication for opioid use disorder, primary health care

## Abstract

**Background:**

Low-threshold care (LTC) practices for prescribing medication for opioid use disorder (MOUD) systematically remove treatment barriers, increasing access to lifesaving MOUD. Despite its promise, LTC operationalization is unclear and heterogeneous, and lacks standardized measures.

**Objective:**

To develop and test LTC composite measures as useful predictors of implementation outcomes.

**Design:**

This prospective cohort study was embedded within a California state MOUD practice change collaborative involving safety-net primary care clinics.

**Participants:**

Data were collected at baseline, midpoint, and endpoint from 20 clinics.

**Intervention:**

Clinics received a multifaceted implementation–support package designed to improve MOUD delivery.

**Main Measures:**

Four LTC scales (LTC12, LTC5, LTC3, LTC2) were developed and tested using team-reported one to five Likert items. Implementation outcomes included Reach (monthly new MOUD patients), Retention (monthly new MOUD patients engaged in treatment after initial diagnosis), and Adoption (active MOUD prescribers). Analyses included repeated-measures ANOVA for LTC change and Poisson GEE for incidence rate ratios, adjusting for panel size, medically underserved area designation, and time.

**Key Results:**

Clinics showed significant improvements in LTC scores over time. The LTC12 scale demonstrated the largest effect size (*d* = 1.18, *p* = .003). A 1-point increase on the LTC3 index was associated with a 37% increase in new patients receiving MOUD (IRR = 1.37, 95% CI [1.01,1.86], *p* = .047). A 1-point increase on LTC2 was associated with a 24% increase (IRR = 1.24, 95% CI [1.01,1.53], *p* = 0.049).

**Conclusions:**

Our findings provide preliminary empirical support for a replicable measure of LTC in primary care settings. Longer scales showed greater internal consistency and sensitivity to change, while brief scales predicted patient reach outcomes. These measures may be useful for clinical programs to gauge the extent to which their MOUD services align with low threshold care principles and to guide quality improvement efforts. Future research should validate these scales in larger, diverse cohorts and test causal impact.

**Supplementary Information:**

The online version contains supplementary material available at 10.1007/s11606-025-10112-9.

## INTRODUCTION

Low-threshold care (LTC) approaches for prescribing medication for opioid use disorder (MOUD) aim to systematically remove or minimize barriers to treatment, thereby increasing access to lifesaving pharmacotherapy.^1^ Expanded access to MOUD is needed as the opioid overdose crisis continues in the USA with over 87,000 drug-related overdoses in 2024.^2^ Yet only one in five adults with opioid use disorder receive MOUD.^3^ Rooted in lessons from HIV, harm reduction, and chronic-disease management, LTC embraces person-centered, risk-reduction principles that depart sharply from abstinence-oriented ideologies that have long dominated addiction care.^4,5^

Core LTC components include rapid or same-day buprenorphine prescription, walk-in hours, telehealth integration, no mandatory counseling, minimal urine–drug screening, and a non-punitive stance toward concurrent substance use.^1,6^ Adoption of LTC models may improve patient outcomes. LTC practices, such as same-day prescription and lack of counseling requirements, are associated with increased access to MOUD.^7,8^ LTC practices are acceptable to patients who report appreciating the non-judgmental regard and accessibility of LTC models (Martinez et al., 2024; Lowenstein et al., 2023). Providing LTC within primary care also offers the opportunity to deliver other necessary primary health services, and integrating LTC in primary-care settings has the potential to increase the reach of LTC models. In a large, multi-site study, 36% of primary care patients met the DSM-5 criteria for a SUD (Wu et al., 2017). Thus, increasing uptake of LTC MOUD models in primary care has the potential to dramatically reduce mortality rates associated with hazardous opioid use and improve public health.

Despite its promise, it is unknown if primary care clinics are able to effectively implement LTC practices due to multilevel barriers.^9,10^ Primary care clinics may struggle to implement components of LTC, such as same-day buprenorphine prescription due to lack of providers with expertise or limited scheduling flexibility for walk-in appointments. Staff may lack experience and training in SUDs, which may lead to stigmatizing attitudes toward patients (Van Boeklel et al., 2013). Primary care-based buprenorphine providers may have lower volumes of patients with OUD than specialized SUD treatment programs; thus, they may have less comfort in providing buprenorphine via telehealth or responding to ongoing substance use.^11,12^

Currently, standardized measures and tools for LTC operationalization are not available or underutilized. Studies commonly use ad hoc process indicators (e.g., “initiated within 72 h,” “drop-in available”) rather than a standard measure.^13,14^ Systematically measuring LTC is an important step for research, practice, and implementation.^6^ Since LTC is inconsistently defined, little is known about how many primary-care clinics adopt its key components or sustain them over time. MOUD practice change models with scalable and measured outcomes are effective ways to increase prescribing and retention in primary care.^15^ In the absence of a measure of LTC, no data are available exploring differential outcomes (e.g. patient reach and retention) as a function of LTC or less LTC approaches. Standardized measures of LTC are thus needed to operationalize and support the scale-up of LTC approaches for MOUD in primary care settings.

The present study introduces and explores the *preliminary utility* of four tiered LTC composite measures (LTC-12, −5, −3, −2) derived from the Integrating Medications for Addiction Treatment in Primary Care – Equity Version (IMAT PCE) as predictors of implementation outcomes across 20 primary care clinics.^16^ These findings will provide the initial empirical evidence for deploying a common and replicable measure of LTC in primary care and other health care contexts. Using longitudinal data from 20 California primary-care clinics, we examine changes in LTC scores over 16 months. The primary objective was to examine whether higher LTC scores predict improved implementation outcomes (i.e., patient reach, retention, and provider adoption).

This study is a secondary analysis of data from the Addiction Treatment Starts Here (ATSH) practice change program.^17,18^ The ATSH program was designed for federally qualified health center primary care clinics in California that were interested in starting up or scaling up MOUD practice. Each clinic set their own goals in collaboration with an ATSH coach who was an expert in addiction medicine, addiction psychiatry, and/or process improvement. Importantly, the project did not explicitly focus on LTC implementation; each clinic established its own improvement goals. Therefore, the present study examines whether LTC practices emerged during the broader implementation of the MOUD practice change program.

## METHODS

### Study Design and Timeline

We conducted a longitudinal implementation study of 20 safety-net primary care clinics in California participating in the Addiction Treatment Starts Here (ATSH) program; additional information on the ATSH program can be found in previous studies.^17^ ATSH is an MOUD practice change program to help teams integrate and improve the delivery of MOUD. While ATSH support may have facilitated LTC practices (e.g., coaching on reducing barriers, peer sharing of rapid-access protocols), clinics were not systematically instructed to adopt LTC principles. Its overarching goal was to reduce substance use treatment inequities through making MOUD more accessible and strengthening partnerships between clinics and their community partners. To achieve this goal, the ATSH program used 4 implementation strategies: (a) audit and feedback, which involved routine collection and reporting of performance data back to clinics to inform ongoing quality improvement efforts; (b) a learning collaborative that included structured workshops on organizational change strategies and problem-solving; (c) external facilitation involving on-site coaching visits to support leadership, staff, and providers; and (d) educational didactic webinars, peer forums, and structured site visits to share educational resources and enhance peer learning.^17^ Data collection for the present study occurred between February 2023 and June 2024 and consisted of three timepoints (baseline, midpoint, and endpoint).

### Sample Selection and Study Population

A total of 20 primary care clinics that applied to participate were enrolled in the MOUD practice change program and were screened for eligibility with the following criteria: (1) provided care in the State of California, (2) provided comprehensive primary care services to underserved populations, (3) met the definition for non-profit/tax-exempt status under 501(C)(3) or governmental/tribal entity, (4) had an established MOUD program that had been in operation for not less than one year.

Participating clinics also reported baseline data on clinic characteristics including geographic location, medically underserved designation (i.e., medically underserved area or population), organization size (categorized as 0–14,999, 15,000–59,999, or ≥ 60,000 patients), organization type (Federally Qualified Health Center (FQHC), FQHC look-alike, ambulatory care clinic, Indian Health Services clinic, or rural health clinic), and payer mix (Medicaid, Medicare, dual eligible, private insurance, and uninsured rates).^19^ FQHCs are nonprofits or public organizations receiving funding from the Health Resources and Services Administration,^20^ and FQHC look-alikes are community health centers that provide primary care and are eligible for FQHC reimbursement structures.^21^ Data were reported using the Research Electronic Data Capture (REDCap) portal, hosted and developed by the Stanford evaluation team.

### Low Threshold Care Index

The primary predictor is the LTC index, which was derived from selected items of the validated Integrating Medications for Addiction Treatment in Primary Care-Equity (IMAT-PCE) index.^16^ The IMAT-PCE is a validated, team-based instrument designed to assess MOUD implementation across diverse clinical settings and has a strong internal consistency (Cronbach’s α = 0.89). The IMAT-PCE was administered at three time points (baseline, midpoint, and endpoint) by clinic staff in collaboration with trained research personnel. While subsequent versions of the IMAT-PCE have incorporated LTC-related items, their predictive validity remains unestablished.

To identify essential aspects of LTC that predict patient outcomes and to provide programs and researchers with greater measurement flexibility in future work, we developed multiple LTC indices of varying lengths. First, we selected items from the full 59-question IMAT-PCE consistent with LTC approaches based on literature review, federal recommendations for defining LTC practices,^6^ and expert consensus. Four LTC indices of varying lengths were developed a priori to balance comprehensiveness and implementation feasibility. The 12-item scale was derived from the IMAT-PCE based on guidelines, expert consensus recommendations, and best practice information. Three shorter indices (LTC-5, LTC-3, and LTC-2) were subsequently created by the clinical co-author team. The 5-item version was developed to streamline clinic assessments while still preserving sufficient LTC domain representation. The 3-item and 2-item versions prioritized items that were most theoretically proximal to patient access as assessed by clinical expertise and literature review. Using the selected items, we created four different LTC indices, ranging from two to twelve items in length (Table 1). Items were summed with equal weighting to create a total LTC composite score, with higher scores indicating better integration of LTC approaches. Although there are multiple ways to measure and interpret composite index scores, the “indicator average” method used in this study is suitable for comparing healthcare organizations with heterogeneous populations.^22^ We then assessed internal consistency for each of the LTC indices. The LTC-12 demonstrated good internal consistency (α = 0.85, 95% CI: 0.80–0.91), while shorter composite measures showed progressively lower internal consistency: LTC-5 (α = 0.63, 95% CI: 0.47–0.78), LTC-2 (α = 0.59, 95% CI: 0.38–0.79), and LTC-3 (α = 0.43, 95% CI: 0.17–0.68). The lower internal consistency of shorter indices was expected given the smaller number of items measuring diverse practices.^23^ No previous study has utilized these LTC items as a composite measure to assess their predictive validity.

**Table 1:** Low Threshold Care Index Items from the Integrating Medications for Addiction Treatment in Primary Care – Equity (IMAT-PCE) index

Item description	LTC-12	LTC-5	LTC-3	LTC-2
Three components are performed for all patients using medications for OUD: Withdrawal symptoms are evaluated, side effects are discussed, and comfort medications to treat opioid withdrawal are made available	X			
Patients choosing medications for OUD can be started within 72 h (buprenorphine) or nearly after 72 h (ER naltrexone injection)	X	X	X	X
Using a protocol clear to both staff and patients, eligible patients can start the medication either in-home or in-office	X	X		
Patients are neither encouraged nor required to taper or discontinue the medication after a certain period of time or once stabilized or with improved functioning	X			
There is easy access to MAT services, including drop-in availability, rapid phone or text follow-up to patient inquiries or requests	X	X	X	X
MAT and other behavioral health services are offered through both telehealth (real-time audio-visual) and telephone modalities	X	X	X	
An outreach procedure exists for patients who have not made appointments or about whom there is clinical concern (phone or home visit)	X			
Clinic leadership engages in regular meetings with other organizations in the geographic region (patient-centered medical neighborhood) to troubleshoot, improve communication, and strengthen the network of care	X			
X-waivered prescriber(s) onsite to prescribe medications for OUD	X			
Nursing or pharmacist personnel are onsite to manage medications for OUD and nursing-related needs of patients; a nurse or pharmacist care manager model is used to perform activities during patient visits either in individual or group formats; there is coordination of care with other healthcare providers; patient and family education is provided	X			
All staff (clinical and non-clinical) have completed training in empathy and stigma reduction for persons with substance use disorders	X	X		
Care teams at the clinic are trained on how to communicate with patients and address trauma, protective factors, adverse life experiences (ACEs), toxic stress, and other trauma-related health risks in the primary care setting	X			

*IMAT PCE*, Integrating Medications for Addiction Treatment in Primary Care Equity; *LTC*, low threshold care; OUD, opioid use disorder; *MAT*, medication-assisted treatment; *ER*, extended-release; *ACEs*, adverse childhood experiences

We then looked at the association between the four different LTC indices and implementation outcomes for the 20 participating clinics. We identified implementation outcomes using the Reach, Effectiveness, Adoption, Implementation, Maintenance (RE-AIM) framework.^24^ The primary outcome was *new patient reach* defined as the number of patients newly initiating MOUD (buprenorphine or extended-release naltrexone) per month. This refers to the number of patients who were newly started on MOUD or who re-started MOUD during the specified month. Secondary outcomes included *patient retention* and *provider adoption*. Patient retention was defined as the number of patients who received two or more outpatient follow-up visits within 34 days of MOUD initiation. Thirty-day retention has been reported to be positively associated with six-month MOUD retention rate, which is an expert-consensus benchmark of MOUD quality of care as presented in the addiction care cascade.^25^ Provider adoption was operationalized as the number of prescribers who have prescribed MOUD (buprenorphine or naltrexone) to at least 1 patient during the month.

### Statistical Analysis

We used descriptive statistics to characterize baseline clinic characteristics. For our main predictors (LTC indices), we used repeated-measures ANOVA to test average changes in each LTC index across three time points (i.e., baseline, midpoint, and endpoint). We also calculated Cohen’s d effect size for the baseline to endpoint comparisons. A post-hoc power analysis indicated that with 20 clinics over three timepoints, we were able to detect small to medium effect sizes in our analyses. While this represents a limitation for the analyses, our sample size aligns with implementation science standards for multi-site primary care studies and was determined by program participation rather than prospective power calculations.

For our main analysis, we estimated the longitudinal associations between LTC scores and our main study outcomes with Poisson GEEs (exchangeable correlation), adjusting for clinic-level covariates (timepoint, organization size, and medically underserved designation).^26,27^ Our goal was theory generation rather than producing precise population estimates, so we prioritized a model that provided stable, interpretable estimates under small sample conditions. Poisson GEE with robust (sandwich) variance is well-established for exploratory implementation studies where dispersion is secondary to directional inference.^26,27^

We conducted separate Poisson GEE models for each of the four LTC indices (LTC-12, 5, 3, 2) as the main predictor with each of the three implementation outcomes (reach, retention, and adoption) and included covariates. We evaluated 12 primary comparisons (four LTC indices × three outcomes); we did not perform any corrections for multiple comparisons due to the *exploratory and descriptive nature of our study*. To understand the differential predictive utility of the LTC indices, we conducted a post-hoc descriptive analysis examining the proportion of clinics achieving meaningful implementation (defined as score ≥ 4 on a 5-point scale) for each item by endpoint (Supplementary Table S1). Missing data for patient retention were handled using single imputation (< 2%), and we had complete data for other variables.^28^ Analyses were completed using R Studio version 4.3.^29^ Study procedures were approved by Stanford IRB as exempt status due to de-identified aggregate data.

## RESULTS

Our sample consisted of 20 clinics with diverse organizational and patient population characteristics (Table 2). The majority were designated as FQHCs (70%), located in urban areas (95%), with small (< 14,999) patient panel sizes (60%) and without medically underserviced status (70%). At baseline, clinics had an average of 4 (range 0–24) active MOUD prescribers and an average of 64 (range 0–420) patients prescribed MOUD, with the majority (64%) insured by Medicaid, followed by Medicare (12%), or uninsured (12%).

**Table 2:** Descriptive Statistics for Clinic Characteristics and Patient Demographics at Baseline (*N* = 20)

Clinic characteristic	*N* (%)
Population density	
Urban/metropolitan	19 (95)
Rural (FORHP-designated)	1 (5)
Primary care shortage	
No medically underserved area designation	14 (70)
Medically underserved designation	6 (30)
Primary care clinic designation	
Federally Qualified Health Center (FQHC)	12 (60)
FQHC look-alike	1 (5)
Ambulatory care clinic owned and operated by a public hospital	6 (30)
Indian Health Service clinic	1 (5)
Organization size	
Small size (0–14,999 patients)	12 (60)
Medium size (15,000–59,999 patients)	5 (25)
Large size (60,000 or more patients)	3 (15)
MOUD indicators, mean % (range)	
Number of all active MOUD prescribers	4 (0—24)
Number of all patients prescribed MOUD	64 (0—420)
Patient insurance type, mean % (range)	
Medicaid (%)	64 (10—92)
Medicare (%)	12 (1—34)
Dual eligibility (%)	6 (0—40)
Private insurance (%)	7 (0—27)
Uninsured (%)	12 (0—23)

*FORHP*, Federal Office of Rural Health Policy; *FQHC*, Federally Qualified Health Center; *MOUD*, medication for opioid use disorder

**Figure 1:**
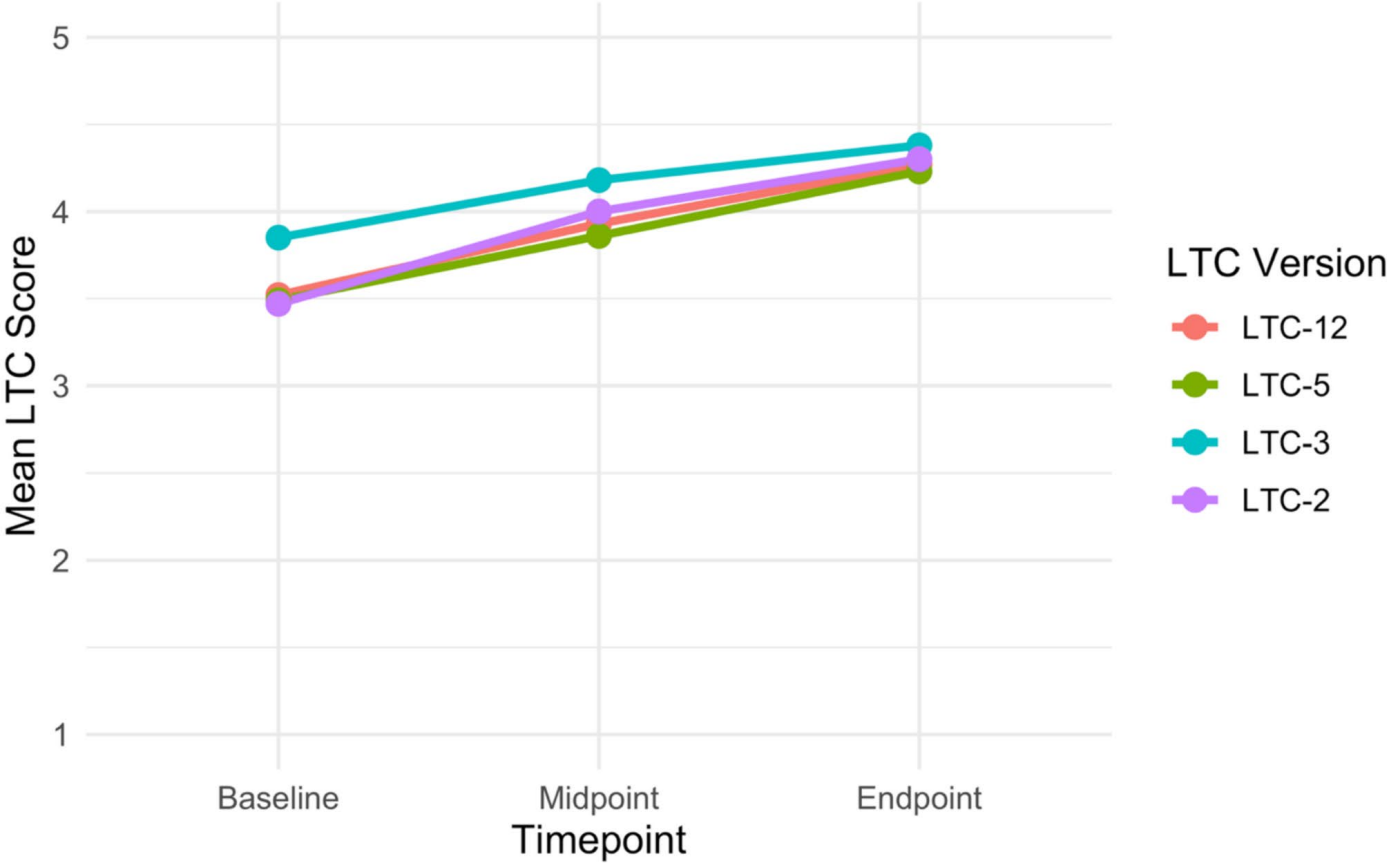
Mean Low Threshold Care (LTC) scores over time by index version. Displayed are the mean scores for the four different Low Threshold Care (LTC) index versions (LTC-12, LTC-5, LTC-3, and LTC-2) across three time points: baseline, midpoint, and endpoint. Each colored line represents a distinct LTC index version, showing how the average score for each index changed over the study period. Higher scores indicate greater integration of LTC practices.

**Figure 2:**
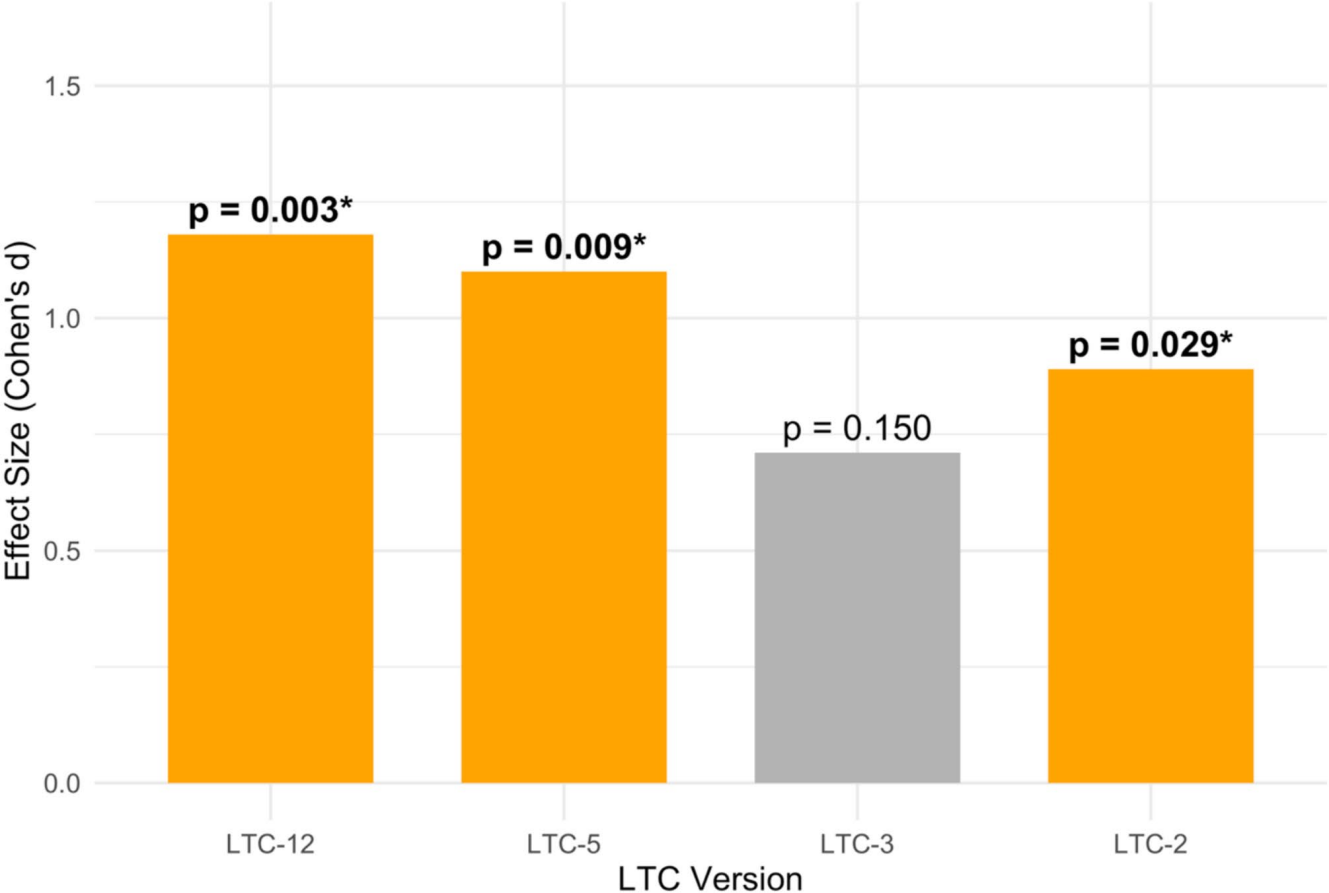
Effect sizes (Cohen’s* d*) for changes in Low Threshold Care (LTC) indices from baseline to endpoint. This bar chart illustrates the effect sizes (Cohen’s *d*) for the observed changes in each of the four Low Threshold Care (LTC) index versions from baseline to endpoint. The height of each bar represents the magnitude of the change, with numerical labels indicating the specific Cohen’s *d* value. Asterisks (*) denote statistical significance (*p* < 0.05), and bold text for the* p*-value label further highlights significant findings. A larger Cohen’s* d* indicates a more substantial improvement in the LTC index score.

**Figure 3:**
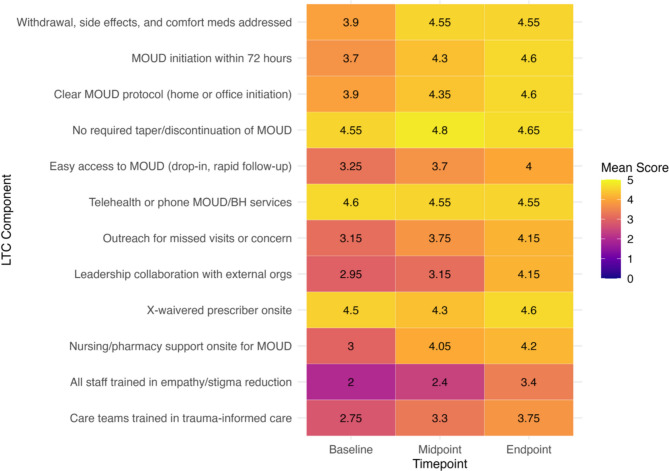
Mean item-level scores across 12 Low Threshold Care components. This heatmap presents the average scores for each of the 12 individual components of the Low Threshold Care (LTC-12) index across three time points: baseline, midpoint, and endpoint. The color of each cell within the heatmap represents the mean score for a specific LTC component at a given timepoint, with a gradient from darker (lower scores) to lighter (higher scores). The numerical mean score is also displayed within each cell, offering a detailed view of the implementation progress for each LTC component over time.

We present the average changes in the four LTC index scores over time in Table 3; Fig. 1. The effect sizes for all four indices ranged from medium (0.71) to large (1.18), as measured by Cohen’s d (Fig. 2); they illustrate greater change in LTC scores for the longer LTC versions. The average LTC index score increased significantly over time for the LTC-12 (*p* = 0.003), LTC-5 (*p* = 0.009), and LTC-2 (*p* = 0.029); the LTC-3 index change was not significant. All index score improvements were observed by the midpoint and further increased by the endpoint. Figure 3 displays mean item-level scores across the 12 LTC components at three timepoints (baseline, midpoint, endpoint).

**Table 3:** Change in Low Threshold Care Index Scores from Baseline to Endpoint with Effect Sizes (*N* = 20)

LTC Index Version*	Baseline Mean (SD)	Midpoint Mean (SD)	Endpoint Mean (SD)	Change mean difference	*P*-value	Effect size (Cohen’s *d*)
LTC-12	3.52 (0.80)	3.93 (0.46)	4.27 (0.40)	0.75	0.003‡	1.18 (0.51–1.85)
LTC-5	3.49 (0.88)	3.86 (0.49)	4.23 (0.37)	0.74	0.009‡	1.10 (0.43–1.76)
LTC-3	3.85 (0.91)	4.18 (0.54)	4.38 (0.54)	0.53	0.15	0.71 (0.07–1.35)
LTC-2	3.47 (1.13)	4.00 (0.63)	4.30 (0.68)	0.83	0.029†	0.89 (0.24–1.54)

^*^LTC, Low threshold care; *SD*, standard deviation; *CI*, confidence interval

^*†*^*p* < 0.05; ^*‡*^*p* < 0.01

The incidence rate ratios for the Poisson GEE models are shown in Table 4. Higher scores on the LTC3 and LTC2 indices were associated with a statistically significant increase in the number of new patients who received MOUD (p < 0.05). Specifically, for each one-point increase on the LTC3 index, the number of new patients would be expected to increase by 37% (IRR 1.37, 95% CI: 1.01–1.86, *p* = 0.047). For each one-point increase on the LTC2 index, the number of new patients would be expected to increase by 24% (IRR 1.24, 95% CI: 1.01–1.54, p-value = 0.049). No statistically significant associations were observed for patient retention or provider adoption after covariate adjustment.

**Table 4:** Incidence Rate Ratios from Poisson GEE Models Predicting Implementation Outcomes by Low Threshold Care Index Scores

LTC Index	Implementation outcome	IRR*	95% CI (IRR)	*p*-value
LTC12	Reach: new patients	1.09	(0.75–1.59)	0.660
LTC12	Adoption: number of MOUD providers	0.93	(0.64–1.34)	0.686
LTC12	Retention	1.07	(0.67–1.70)	0.779
LTC5	Reach: new patients	1.13	(0.83–1.52)	0.440
LTC5	Adoption: number of MOUD providers	1.34	(0.96–1.87)	0.088
LTC5	Retention	1.12	(0.67–1.86)	0.679
LTC3	Reach: new patients	1.37	(1.01–1.86)	0.047†
LTC3	Adoption: number of MOUD providers	1.22	(0.81–1.86)	0.343
LTC3	Retention	1.08	(0.74–1.58)	0.697
LTC2	Reach: new patients	1.24	(1.01–1.53)	0.049†
LTC2	Adoption: number of MOUD providers	1.19	(0.97–1.46)	0.086
LTC2	Retention	1.04	(0.80–1.35)	0.781

Each model controls for clinic panel size, medically underserved designation, and timepoint

*IRRs*, incidence rate ratios from Poisson generalized estimating equation models with an exchangeable correlation structure; *MOUD*, medications for opioid use disorder

^†^Significant at *p* < 0.05

The post-hoc descriptive analysis showed that most LTC indicators showed ceiling effects, with 80% of clinics scoring ≥ 4 by the final wave; this was particularly true for items related to MOUD initiation protocols (i.e., rapid initiation, home or office induction, and withdrawal management). The comprehensive LTC-12 average score was 4.27 (SD = 0.4), which suggests restricted variance in the longer composites. In contrast, although 75% of clinics scored ≥ 4 on both items in the LTC-2 index (rapid MOUD initiation and drop-in access), the remaining heterogeneity between clinics was sufficient to retain predictive utility. Items in the shorter LTC-2/LTC-3 indices showed greater variability than those in longer composites (Supplementary Table 1).

We conducted sensitivity analyses using negative-binomial GEE models with identical correlation structures and covariates. However, several models failed to converge due to sparse counts and limited cluster sizes; when convergence was achieved, incidence rate ratios were directionally consistent with our main model. These outcomes support the use of Poisson GEE with robust variance for the main analyses.

## DISCUSSION

Our findings suggest that the tiered LTC indices have predictive utility for patient access to MOUD and are sensitive to changes over time in a large implementation trial. Clinics showed significant increases in LTC scores over time after receiving MOUD implementation support, and these changes predicted improvements in patient reach. LTC scores did not predict significant changes in measures of retention and provider adoption. Various stakeholders can use these indices to fit their specific context and needs. This is the first study to demonstrate an association between LTC and improved patient access to MOUD in primary care settings, using a quantitative definition of LTC.

The post-hoc descriptive analysis suggests that shorter LTC indices were more predictive of patient reach because they captured the items with sufficient variation and tied directly to patient access (i.e., rapid initiation and drop-in appointments). These items retained meaningful between-clinic variation even as overall MOUD implementation matured. Longer indices included items that had limited variability by endpoint or were distal to patient reach, which potentially reduced their predictive power. This pattern aligns with our intentional design of tiered indices to allow flexibility based on contextual factors such as implementation stage and partner priorities. High endorsement (e.g., 95% scoring ≥ 4 on rapid initiation) likely reflects measurement ceiling rather than diminished clinical importance.

Our findings that LTC predicted improvements in patient reach but not retention or provider adoption is consistent with the theoretical mechanisms of LTC models. Overall, LTC practices like rapid initiation, telehealth, and drop-in access are designed to directly remove barriers to treatment access; this potentially explains the positive association with increased patient reach.^7,30,31^ The lack of significant findings for retention and adoption likely reflects multiple mechanisms and is consistent with the current literature, which contains mixed findings. Paradoxically, LTC may simultaneously improve retention through flexible and non-punitive care while also engaging patients with greater structural barriers that face increased retention challenges.^8,32^ Individuals in low threshold programs may thus have multiple treatment “episodes” as opposed to continuous retention in treatment.^33–35^ Future work must find ways to differentiate between those two potentially conflicting patterns.

The lack of significance with provider adoption suggests that low-threshold clinical practices may not, by themselves, address provider-level barriers such as clinical training gaps, stigma, regulatory oversight, etc..^36,37^ The removal of the X-waiver requirement in 2023 did not substantially increase buprenorphine prescribing rates among eligible providers, which illustrates the complexity of provider-level barriers.^38^ Future work should examine whether targeted interventions for providers (i.e., stigma reduction training) may act as important mediators or moderators to large-scale LTC implementation success.^39^

Composite measures that combine multiple practice items, such as the current LTC index, can be useful for clinicians and/or payers to measure quality improvement.^40^ A particular strength of the current tiered LTC index is its utility in multiple healthcare contexts. LTC indices can function as a practical tool for system and practice design, continuous quality improvement, implementation planning, and workforce education.^16^ Regional dashboards could track LTC scores and respond to threshold changes (e.g., scores less than 4/5) through continuous monitoring and integrated EHR reports.^41^ Clinic scores can reveal organizational deficiencies, which can be targets for staff training and specific implementation strategies. LTC measurement thresholds can also be used as payer incentives as part of a cascade of care.^25^ Incorporating LTC measures into diverse implementation strategies and health systems infrastructure can thus improve MOUD delivery and lead to better patient outcomes.

Findings from our study align with recent evidence indicating that LTC MOUD models have positive impacts on patient and implementation outcomes in diverse healthcare settings. Our findings coincide with recent policy shifts that remove regulatory barriers to buprenorphine access, such as the removal of counseling requirements for patients at the state level and the federal X-waiver for prescribers, which removed training barriers.^42^ Telehealth requirements have also been loosened to provide pandemic flexibility, and evidence suggests that these changes increased prescribing of MOUD and have been found acceptable by both patients and providers.^43,44^ These regulatory changes underscore the timely need for scalable LTC evaluation methods to assess what level or mix of policy interventions (e.g., X-waiver elimination and telehealth flexibilities) increase access to MOUD.

Our findings also contribute to the body of work on integrating buprenorphine in primary care settings. Despite expanded prescribing authority following X-waiver removal and federal policy changes, levels of primary care-based buprenorphine prescribing have largely plateaued since 2018.^45,46^ Recent growth has been driven primarily by nurse practitioners and physician assistants rather than primary care physicians.^47^ Since the present study was part of a large implementation trial to increase buprenorphine prescribing in primary care, it was encouraging to see LTC scores increase over time. This was notable even though there was no LTC-specific curriculum provided in the parent study, though clinics were able to request coaching on LTC topics. These positive findings demonstrate the continued promise of integrating buprenorphine services in primary care settings. Providing technical assistance and support to specifically enhance LTC delivery in primary care settings could be an important area for future research.

Our study has several limitations that warrant cautious interpretation of our findings. First, the exploratory cohort design and self-reported LTC items may introduce measurement (e.g., social desirability) bias. Additionally, our post-hoc descriptive results should be interpreted cautiously. We present ceiling effects using non-empirical cutoffs (e.g., scores ≥ 4) to illustrate variability patterns rather than to test inferential hypotheses. Clinical performance on quality indicators can also be hard to interpret.^22^ The small (*n* = 20), California safety-net clinic sample limits generalizability to other states, and we may have been underpowered to detect small to medium effect sizes. Importantly, our exploratory analysis plan and multiple comparisons increased the chance of Type I error (false positive); wide confidence intervals suggest that results should be interpreted cautiously. Our limited number of clinics precluded the use of more flexible models that directly model dispersion (e.g., negative-binomial). Comprehensive psychometric validation was outside the scope of this initial exploratory study. Our findings near the p < 0.05 threshold should be viewed as signals warranting confirmation; these findings should be interpreted as exploratory in nature and used for hypothesis generation. We cannot establish the specific implementation strategies that drove LTC improvements. These may have included the clinic-directed improvement targets developed with coaches. Additionally, this was a secondary analysis of data collected for other purposes, which constrained our ability to comprehensively measure all dimensions of LTC.

In light of these limitations, validating LTC measures in larger, more diverse cohorts will strengthen their utility for MOUD implementation. Future studies should validate these findings using alternative modeling approaches and directly test LTC-focused change strategies in primary care settings. Larger studies can clarify meaningful thresholds of LTC practice integration.^22^ External validation in diverse states, private systems, and controlled trials is warranted to empirically test whether enhancing LTC scores causally improves patient outcomes. Incorporating LTC items into health system quality dashboards may help accelerate MOUD scale-up.

## CONCLUSION

We provide four indices of differing lengths as potential standardized measures of LTC practice integration. Across 20 safety-net clinics, average LTC scores rose significantly over 16 months of implementation support. Longer indices were more sensitive to LTC practice change as evidenced by larger effect size differences, but shorter indices had better utility in predicting implementation outcomes. Each 1-point increase on the LTC-3 and LTC-2 indices was associated with 37% and 24% more newly treated patients without significant change in patient retention or prescriber adoption. Clinical programs and researchers can choose the index that best fits their practice context and data gathering needs. These LTC measures may be useful to clinical programs in determining the extent to which their MOUD services meet the definition of low-threshold care and guide quality improvement efforts.

## Supplementary Information

Below is the link to the electronic supplementary material.

Supplementary Material 1 (DOCX 16.7 KB)

## Data Availability

The datasets generated and analyzed during the current study are not publicly available due to the sensitive nature of the primary care clinic performance data but are available from the corresponding author on reasonable request.
