# Supplementary material for: Implementation Outcomes of Low Threshold Care for Persons with Opioid Use Disorders
Source: J Gen Intern Med. 2026 Jan 14;41(9):2437–47. doi: 10.1007/s11606-025-10112-9 (PMC12988830; doi:10.1007/s11606-025-10112-9)
Supplement: Supplementary file 1 — Supplementary Material 1 (DOCX 16.7 KB) [file 11606_2025_10112_MOESM1_ESM.docx]

| Table S1. Low Threshold Care Item and Composite Score Distribution at Endpoint | |
| --- | --- |
| Item | Mean Score/Proportion Scoring ≥ 4 |
| **LTC 12 composite summary** |  |
| LTC12 (mean score) | 4.27 / 5 |
| LTC12 mean ≥ 4 | .80 |
| LTC12 – all 12 items ≥ 4 | .05 |
| **Item-level proportions** |  |
| Rapid MOUD initiation (≤ 72 h) | .95 |
| Clear MOUD protocol (Home or office initiation) | .90 |
| No required MOUD taper/discontinuation | .90 |
| Withdrawal and comfort medications addressed | .85 |
| Telehealth or phone-based initiation | .85 |
| X-waivered prescriber available on-site | .85 |
| Nursing/pharmacy support available on-site | .85 |
| Easy access to MOUD (Drop-in, rapid follow-up) | .75 |
| Outreach for missed visits or concern | .75 |
| Leadership collaboration with external organizations | .65 |
| Care teams trained in trauma-informed care | .50 |
| All staff trained in empathy & stigma reduction | .40 |
| **LTC tiered indices** |  |
| LTC2 – all two items ≥ 4 | .75 |
| LTC3 – all three items ≥ 4 | .65 |
| LTC5 – all five items ≥ 4 | .20 |

Note. Values reflect the proportion of clinics scoring ≥ 4 on each item or composite. These proportions are descriptive and highlight ceiling effects that reduce variability in longer indices (LTC-12, LTC-5). Shorter indices (LTC-2, LTC-3) retained greater variability.
