# Supplementary material for: Implementation Outcomes of Low Threshold Care for Persons with Opioid Use Disorders
Source: J Gen Intern Med. Author manuscript; Available in PMC 2026 Mar 15. (PMC12988830; doi:10.1007/s11606-025-10112-9)
Supplement: Supplementary file 1 [file NIHMS2147280-supplement-S1.pdf]

Table S1. Low Threshold Care Item and Composite Score Distribution at Endpoint

Item	Mean Score/Proportion Scoring ≥ 4
**LTC 12 composite summary**	
LTC12 (mean score)	4.27 / 5
LTC12 mean ≥ 4	.80
LTC12 – all 12 items ≥ 4	.05
**Item-level proportions**	
Rapid MOUD initiation (≤ 72 h)	.95
Clear MOUD protocol (Home or office initiation)	.90
No required MOUD taper/discontinuation	.90
Withdrawal and comfort medications addressed	.85
Telehealth or phone-based initiation	.85
X-waivered prescriber available on-site	.85
Nursing/pharmacy support available on-site	.85
Easy access to MOUD (Drop-in, rapid follow-up)	.75
Outreach for missed visits or concern	.75
Leadership collaboration with external organizations	.65
Care teams trained in trauma-informed care	.50
All staff trained in empathy & stigma reduction	.40
**LTC tiered indices**	
LTC2 – all two items ≥ 4	.75
LTC3 – all three items ≥ 4	.65
LTC5 – all five items ≥ 4	.20

Note. Values reflect the proportion of clinics scoring ≥ 4 on each item or composite. These proportions are descriptive and highlight ceiling effects that reduce variability in longer indices (LTC-12, LTC-5). Shorter indices (LTC-2, LTC-3) retained greater variability.
